# Bongani Mayosi, a hero remembered

**Published:** 2018

**Authors:** Ntsekhe Mpiko, Commerford Patrick, Brink Paul, Yusuf Salim

**Affiliations:** Department of Cardiology, Faculty of Health Sciences, University of Cape Town and Groote Schuur Hospital, Cape Town, South Africa; Department of Cardiology, Faculty of Health Sciences, University of Cape Town, Cape Town, South Africa; Department of Internal Medicine, Faculty of Medicine and Health Sciences, University of Stellenbosch and Tygerberg Hospital, Tygerberg, South Africa; Department of Medicine, and Population Health Research Institute, McMaster University, and Hamilton Health Sciences, Hamilton, Ontario, Canada

Shortly after joining the internal medicine rotation at Groote Schuur Hospital, Bongani Mayosi admitted a patient with suspected tuberculous pericarditis to one of the medical wards. Little did he anticipate the reaction of the attending consultant the next day on the post-intake ward round when he let her know that he had added high-dose corticosteroids to the patient’s antituberculosis therapy. Stumped by the simple request to provide evidence for his decision and shocked by the absence of definitive answers when he looked it up, he made a mental note to one day be the person who would provide the answers. Twenty years later, Professor Mayosi had not only published the largest clinical trial of interventions for tuberculous pericarditis in the *New England Journal of Medicine*, but he was widely acknowledged as the world’s foremost authority on the subject.

Mayosi was born and brought up in the Eastern Cape Province of South Africa (Transkei) where his father, the regional district surgeon and his mother, a nurse, inspired his lifetime commitment to patient care, and nurtured his belief that he could be whatever he wanted to be and achieve anything. He often reflected on his early years in rural Transkei where everyone he looked up to, admired and wanted to grow up to be like, looked like him and believed in him. He was grateful to that environment, which he believed had spared him the crippling consequences of self-doubt that is one of the main legacies of apartheid South Africa.

He graduated from St John’s College in Mthatha at the age of 15 years, with six distinctions, and then went on to graduate *cum laude* and at the top of his medical school class from the then University of Natal. When asked to explain the source of his subsequent passion for research, he pointed to, as crucial, the extra year he had taken from his medical degree programme (MB ChB) to study the intricacies of the navicular bone. His MB ChB was followed in quick succession by fellowship of the College of Physicians and formal cardiology training, both at the University of Cape Town (UCT).

In 2001 he returned to Cape Town from Oxford University with a DPhil and the dream of creating a cadre of people and building a programme of clinical research that would be capable of eradicating the unacceptably high burden of neglected diseases of poverty afflicting sub-Saharan Africa. Shortly after that he was appointed and served as professor and head of the Department of Medicine at Groote Schuur Hospital and UCT until 2015.

Whether it was his warm smile, infectious enthusiasm, engaging intellect or irresistible charm, few people who met him were not immediately smitten. Mayosi was endowed with unique combinations of academic brilliance and vision, ambition and humility, and the ability to persuade people around him to believe that they could achieve the near impossible. Among his many mentees and colleagues, he became famous for a number of inspiring ‘Bongani-isms.’ One such -ism, which came to encapsulate so much of who he was and what was important to him, was the idea that we should all strive to ‘lift others as we rise.’ Whether it was individuals or institutions, he believed that there is little point in having lonely islands of success. And importantly, he would argue, Africa’s long-term ability to address the great health-related and other challenges of the day was crucially dependent on making sure we have as many high achievers and people with the requisite skills and ability as possible.

In his role as a global leader in medicine, he managed to strike up and lead critical collaborations with international partners, which allowed him a large number of important scholarly contributions on cardiovascular disease and health at local and international levels. Working with Salim Yusuf of the Population Health Research Institute (PHRI) at McMaster University in Canada, research funding was raised and expertise was developed that allowed for the creation of the Pan-African Investigation of the Management of Pericarditis (IMPI), a multicentre research consortium that conducted many important studies and a trial on tuberculous pericarditis.

Again it was his relationship with partners at the PHRI that led to the establishment of the Global Rheumatic Heart Disease Registry, or REMEDY, which evolved into the first large, multicountry registry and cohort study of 3 000 people with rheumatic heart disease (RHD) across much of the African continent, the Middle East and east Asia. The INVICTUS trial of 4 500 people evaluated antithrombotics in a global registry and cohort study of 20 000 patients in 30 countries, and a Wellcome Trust-funded study worked on the genetics of RHD.

Finally, working with Peter Schwartz, Hugh Watkins and other collaborators from Europe and the United Kingdom, a programme of research on heart muscle disease and novel genes in Africans was established. Through these pioneering programmes, optimal methods for the diagnosis and treatment of tuberculous pericarditis were defined, rheumatic heart disease was put on the global agenda of the World Heart Federation and World Health Organisation, and it led to the discovery of novel genes, which allowed for better understanding of the biological mechanisms of heart disease and fibrosis.

Mayosi’s research output was enormous and included many articles, book chapters and books with multiple citations. His contributions to capacity development and skills output were equally impressive and included numerous individuals who he personally supervised and mentored as academics, clinician scientists and leaders, and even more people whom he inspired, influenced and created opportunities to do the same.

Among his many achievements, he was particularly proud, over the 20 years of service to the organisation, of helping to resurrect the Pan-African Society of Cardiology into a vibrant, active society, and helping to give the organisation a sense of gravitas and purpose, making it fit and able to help tackle Africa’s cardiovascular health priorities.

For his tremendous contribution to society, Mayosi was honoured with numerous awards and prizes during his outstanding career. Among those that he prized most were South Africa’s highest honour, the Order of Mapungubwe in Silver, for excellent contributions to medical science in 2009, the National Science and Technology Foundation – BHP Billiton award 2012 (to an individual for outstanding contribution to science, engineering, technology and innovation through management and related activities over the previous five to 10 years or less), and the National Research Foundation award for transforming the science cohort of South Africa (the award is focused on transforming the science cohort to be more representative of South African demographics).

Importantly, this recognition came as he actively advanced and supported the careers of students and colleagues, irrespective of ethnicity, race, religion or social class. His unique ability to be both ‘colour blind’ and proudly promote transformation and the all-inclusive African-ness was enormously important to a wounded country and it’s institutions of higher learning.

In 2016 Mayosi received the Honorary Fellowship of Wolfson College, University of Oxford (to individuals whom they particularly value and admire for their outstanding distinction in their field, and for the intellectual contribution they have made in the world to areas in which the College has a strong interest). In 2017 he became one of the few Africans inducted to the National Academy of Medicine in the USA.

Bongani remained grounded and humble until the very end. It is fair to say he was respected and revered, and will be remembered as much for who he was as a human being and his qualities of ‘ubuntu’ (humanity towards others) as for what he achieved. He was a peoples’ person who could fit in comfortably almost anywhere with anyone. He developed warm, strong bonds and interpersonal relationships with people at all levels. He was kind and compassionate and cared deeply about the welfare and well-being of others. When asked how it was that he was able to give so much to others, he famously responded by reminding people that ‘the gift of the giving is in the giving’.

He was a devoted, loving family man who met his wife and life partner Professor Nonhlanhla Khumalo on a bus during orientation at medical school when he was 16 years old, and knew immediately that he had found his life partner. Their lifelong bond was palpable and obvious to all who met them. Adorning the walls of his office at work were numerous short, handwritten messages from his three daughters Nosipho, Vuyi and Gugu, who he loved much and were not only incredibly special to him but were his pride and joy.

Bongani was appointed as dean of the Faculty of Medicine at the University of Cape Town in 2016. This appointment coincided with the start of country- and university-wide, student-led unrest and protests, and one of the most difficult periods ever experienced in the higher education landscape. By his own admission, being at the symbolic centre of the tense turmoil that ensued and the target of often intense criticism from all stakeholders was incredibly difficult, given his own value system, beliefs and way of being. Importantly it also took him away from his first love of teaching, training and research, and the grand plans for the Faculty of Health Sciences, country and continent, which he had been developing over the preceding 12 to 15 months, in preparation for becoming dean. No one will ever know or understand how much the events of 2016–2018 contributed to his painful battle with clinical depression, which he eventually succumbed to through suicide on 27 July of this year, but there is little doubt that the turbulent times and environment clearly had a significant impact on him.

Bongani will be sorely missed by all who knew him and knew of him. Among the many legacies he leaves behind are the numerous people, programmes and partnerships, which he inspired and infused with a collective sense of purpose to work towards his dreams of a healthier, wealthier and wiser Africa, capable of defining its own priorities, solving its own problems and being the master of its own destiny.

This article may be reproduced in other medical and scientific journals provided the original source is acknowledged: *Cardiovascular J Afr* 2018; 20(4): 205–206.

